# Supplementary material for: The Multiplex Efficiency Index: unveiling the Brazilian air transportation multiplex network—BATMN
Source: Sci Rep. 2020 Aug 7;10:13339. doi: 10.1038/s41598-020-69974-0 (PMC7414201; doi:10.1038/s41598-020-69974-0)
Supplement: Supplementary file 1 — Supplementary information [file 41598_2020_69974_MOESM1_ESM.pdf]

Supporting information material: The Multiplex Efficiency Index: unveiling the Brazilian Air Transportation Multiplex Network - BATMN

Izabela M. Oliveira,[1,2,*] Laura Carpi,[2] and Allbens Atman Picardi Faria[2,3,4]

[1] *Departamento de Matemática, Centro Federal de Educação Tecnológica de Minas Gerais, CEFET-MG. Av. Amazonas, 7675, Belo Horizonte, MG, Brasil. CEP: 30.510-000.*

[2] *Programa de Pós-Graduação em Modelagem Matemática e Computacional, PPGMMC, CEFET-MG.*

[3] *Departamento de Física, CEFET-MG.*

[4] *Instituto Nacional de Ciência e Tecnologia de Sistemas Complexos, INCT-SC, CEFET-MG.*

NOTE S1. EXAMPLE OF MULTIPLEX EFFICIENCY INDEX CALCULATION

We created two synthetic multiplex structures to illustrate the computation of the $\mathcal{E}$-index (see Fig. S1). As proposed in [1], we calculated the dissimilarities between every pair of layers (shown in matrix LD) and then, the $\mathcal{E}$-index values and the diversity ordering of each structure.

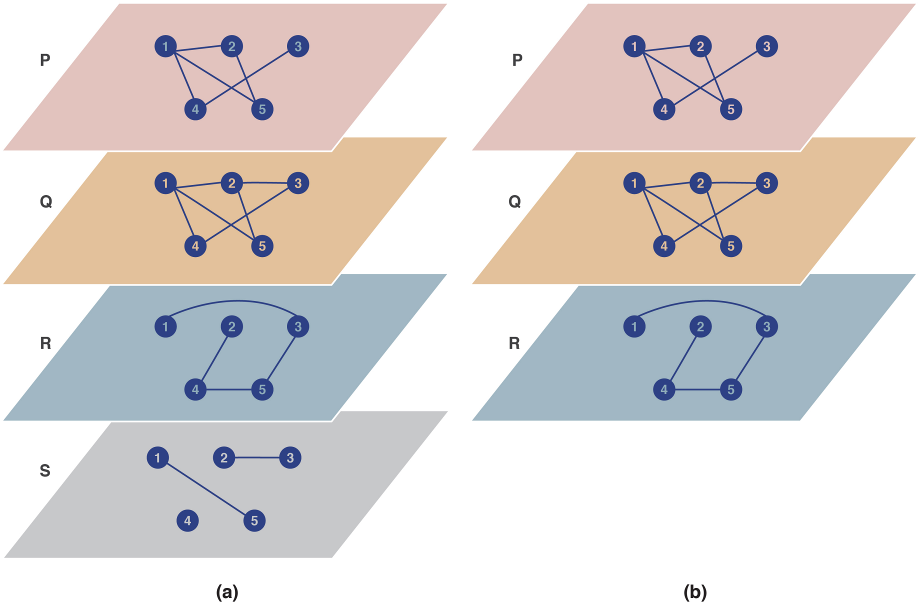

FIG. S1: Illustration of synthetic networks analyzed in experiments 1 and 2. **(a)** Representation of the four-layers multiplex network, used in Experiment 1. As it can be seen in this structure, layers P and Q possess high link overlapping, very similar degree and distance distribution, among other characteristics. The system's diversity ordering $O_D = \{Q, P, R, S\}$ indicates that layer S is the one that contribute the most to the heterogeneity of the multiplex system, as, although it has two redundant links, the degree and distance distribution are very dissimilar to all others, then increasing its $\mathcal{E}$-index ($\mathcal{E} = 0.64$). **(b)** Representation of the three-layers multiplex network used in Experiment 2, obtained from the Experiment 1 network, excluding layer S. This structure is highly redundant in its connectivity patterns. Its diversity ordering $O_D = \{Q, P, R\}$ indicates that R layer is the one that most contributes to the $\mathcal{E}$-index of the system ($\mathcal{E} = 0.49$).

Experiment 1: A four-layer network structure, in which layers P and Q have high link overlap and, consequently, the lowest dissimilarity value (Figure S1-a). The R layer has no overlapping links to the others and presents the greatest dissimilarity for S, as can be seen in the LD_1 matrix.

$$\mathrm{LD}_1 = \begin{array}{c} \\ P \\ Q \\ R \\ S \end{array} \begin{array}{c} \begin{array}{cccc} P & Q & R & S \end{array} \\ \begin{bmatrix} 0 & 0.271 & 0.702 & 0.849 \\ 0.271 & 0 & 0.693 & 0.705 \\ 0.702 & 0.693 & 0 & 0.935 \\ 0.849 & 0.705 & 0.935 & 0 \end{bmatrix} \end{array}$$

Organizing the distances of each layer in ascending order, are:
$\mathcal{D}(P, S_1) = \{0; 0.271; 0.702; 0.849\}$,
$\mathcal{D}(Q, S_1) = \{0; 0.271; 0.693; 0.705\}$,
$\mathcal{D}(R, S_1) = \{0; 0.693; 0.702; 0.935\}$,
$\mathcal{D}(S, S_1) = \{0; 0.705; 0.849; 0.935\}$,
so that S_1 represents the set of layers present in the network at this stage. Thus, the smallest positive value is $\mathcal{D}(P, Q) = 0.271$ which represents the distance between the layers P and Q. Looking at the distance sets of both, we realize that Q has the next shortest distance to another layer and, therefore, the order of diversity receives the first element: $O_D(S) = \{Q\}$, because we add the first value for the recursive calculation of diversity $U = 0.271$.

Next, we eliminate the Q layer and look at the remaining set S_2.
$\mathcal{D}(P, S_2) = \{0; 0.702; 0, 849\}$,
$\mathcal{D}(R, S_2) = \{0; 0.702; 0.935\}$,
$\mathcal{D}(S, S_2) = \{0; 0.849; 0.935\}$,
Now, the smallest LD-value is $D(P, R) = 0.702$. Thus, $U = 0.271 + 0.702$ and, following the lexicographic order, layer P is the closest to another layer, and therefore $O_D(S) = \{Q, P\}$. We now have the remaining S_3 set of layers after deleting P. Thus, we have:
$\mathcal{D}(R, S_3) = \{0; 0, 935\}$,
$\mathcal{D}(S, S_3) = \{0; 0.935\}$.
Clearly, 0.935 is the last amount to be added at $U = 0.271 + 0.702 + 0.935 = 1,908$ and therefore $\mathcal{E} = 0.64$, according to equation (3) of the main text. For the ascending sequence of components of the calculation of $\mathcal{E}$, we observe that the layer R was the "closest" of the set S_2 in the previous step and then $O_D = \{Q, P, R, S\}$. This means that S is the layer that adds highest diversity to the multiplex, while layer Q is the one that less contributes.

Experiment 2: From the system shown in the previous experiment, we eliminated layer S, which was the most significant in calculating the value of diversity. The LD_2 matrix displays the dissimilarity values for the new situation.

$$\mathrm{LD}_2 = \begin{array}{c} \\ P \\ Q \\ R \end{array} \begin{array}{c} \begin{array}{ccc} P & Q & R \end{array} \\ \begin{bmatrix} 0 & 0.271 & 0.701 \\ 0.271 & 0 & 0.693 \\ 0.701 & 0.693 & 0 \end{bmatrix} \end{array}$$

Following the sequence of steps already shown in Experiment 1, we have:
$\mathcal{D}(P, S_1) = \{0; 0.271; 0.701\}$,
$\mathcal{D}(Q, S_1) = \{0; 0.271; 0.693\}$,
$\mathcal{D}(R, S_1) = \{0; 0.693; 0.701\}$,
then, in this first stage, $U = 0.271$ and $O_D = \{Q\}$. Next,
$\mathcal{D}(P, S_2) = \{0; 0.701\}$,
$\mathcal{D}(R, S_2) = \{0; 0.701\}$,
and finally, $U = 0.271 + 0.701 = 0.972$ therefore, $\mathcal{E} = 0.49$ and $O_D = \{Q, P, R\}$. We conclude that layer R is the one that more contributes to the $\mathcal{E}$-index value of the system.

NOTE S2. EVOLUTION OF THE LOCAL MULTIPLEX EFFICIENCY INDEX FOR PRIVATIZED AIRPORTS

We show here the evolution of the local $\mathcal{E}$-index for the airports that were privatized before 2017 [2].

Initially only Natal International Airport (serving the city of Natal - RN and region) is privatized in 2011. Figure S2 shows the evolution of the $\mathcal{E}$-index for this airport.

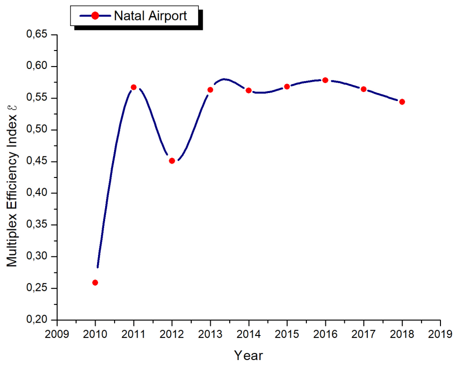

FIG. S2: Evolution of $\mathcal{E}$-index for Natal airport.

In 2012 three airports are privatized:

- Brasilia International Airport,
- São Paulo/Guarulhos International Airport,
- Viracopos International Airport (Campinas - SP).

The $\mathcal{E}$-index for these airports is shown in Fig. S3.

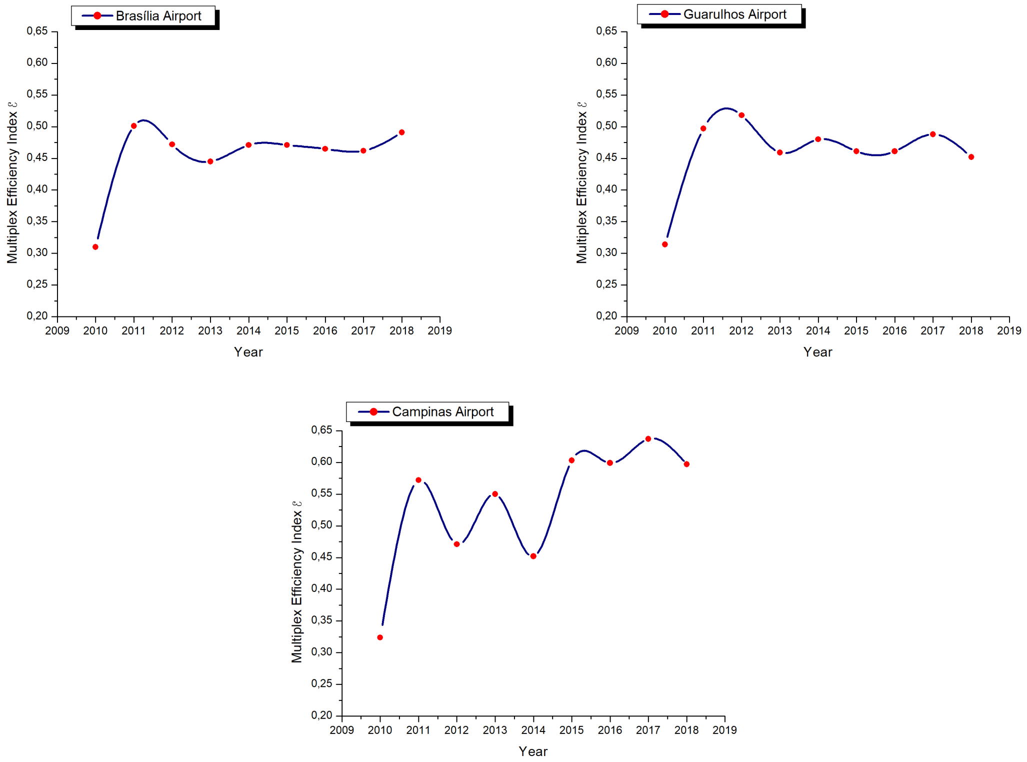

FIG. S3: $\mathcal{E}$-index for Brasilia International Airport, São Paulo/Guarulhos International Airport and Viracopos International Airport.

Finally, two other airports are privatized in 2013 and the Fig. S4 shows the $\mathcal{E}$-index over the period considered for each of these airports:

- Rio de Janeiro/Galeão International Airport, Rio de Janeiro - RJ
- Belo Horizonte/Confins International Airport, MG.

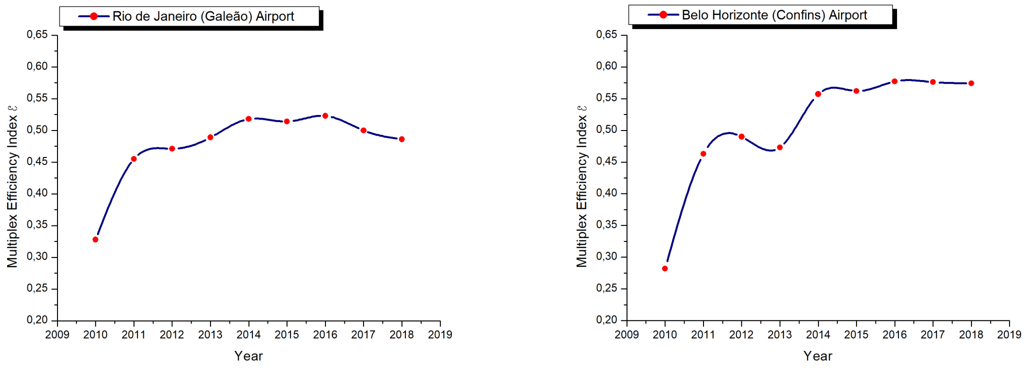

FIG. S4: $\mathcal{E}$-index for Rio de Janeiro/Galeão International Airport and Belo Horizonte/Confins International Airport.

For comparison, we observe the evolution of the $\mathcal{E}$-index for the São Paulo/Congonhas Airport, which was not privatized (Figure S5). This is a hub in the Brazilian air transportation network [3].

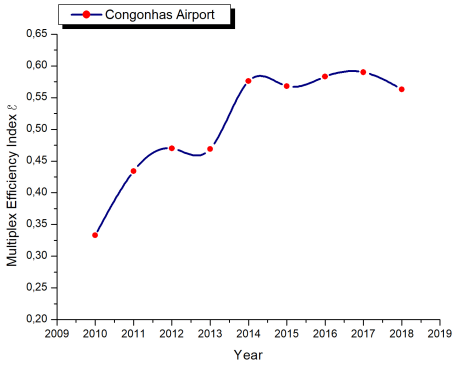

FIG. S5: $\mathcal{E}$-index values to Congonhas Airport.

* izabelamarques@cefetmg.br

[1] L. C. Carpi, T. A. Schieber, P. M. Pardalos, G. Marfany, C. Masoller, A. Díaz-Guilera, and M. G. Ravetti, Scientific Reports **9**, 4511 (2019).
[2] “Dúvidas sobre as concessões?” *Brazilian Government Publications. ANAC - Agência Nacional de Aviação Civil.* `http://www.anac.gov.br/noticias/2011/duvidas-sobre-as-concessoes` (2011).
[3] T. F. Costa, G. Lohmann, and A. V. Oliveira, Research in Transportation Economics **26**, 3 (2010).
